# Treatment of Persons Asphyxiated from Drowning

**Published:** 1845-07-01

**Authors:** 


*Treatment of persons asphyxiated from Drowning.*— James Stewart, M. D. in a communication to the New-York Journal of medicine, for May,

1845, advocates the importance of reserting to external warmth, as the first and most efficient means of restoration in cases of suspended animation from submersion.

The insufflation of the lungs he regards as a secondary measure, and unless practiced with the greatest precaution, as productive of so much injury, that it is worse than useless. He thinks that Electro-Magnetism furnishes an agent which will be equally effectual in all cases in which the respiratory acts are capable of being excited, and being free from the evil consequences of forcible inflation, should be employed to the exclusion of the latter. Before this can be applied, however, external heat should be made use of. Dry heat would be preferable, but it cannot be made so promptly available. The method which will be most readily adopted, is to envelop the body in a large blanket which has been thoroughly saturated with hot water. This can generally be found in the neighborhood, and a single quart will suffice.

Dr. Stewart sustains his views both by pathological considerations, and cases which have fallen under his observation. For these we must refer the reader to his article.

As the application of heat in the manner just mentioned, can be made by any one, and it is of immense consequence to avoid the delay which must often occur before a physician arrives, it is highly desirable that information on the subject should be generally diffused.

